# Editorial: Global excellence in health economics: Africa

**DOI:** 10.3389/fpubh.2024.1460357

**Published:** 2024-09-27

**Authors:** Olatunde Aremu

**Affiliations:** Department of Public Health and Therapies, Faculty of Health Education and Life Sciences, Birmingham City University, Birmingham, United Kingdom

**Keywords:** global collaboration, health economics, public health, health insurance, health service utilization, developing countries, health technology, catastrophic health expenditure

## Introduction

Globally, the cost of providing medical care has continued to rise, due in part to increasing demand for preventive and curative treatments coupled with the high cost of drugs. Sadly, this scenario has exacerbated Africa's unequal access to and utilization of healthcare services. The majority of the African population lives below the poverty level, with many dying from preventable and treatable diseases (1, 2). In many countries in Africa, as in the majority of countries in developing regions of the world, when medical care is available, the cost of accessing it is usually beyond the reach of ordinary people (3, 4). Although sub-Saharan Africa's per capita expenditure on healthcare is one-tenth of the global average, out-of-pocket (OOP) healthcare spending accounts for nearly 30% of current healthcare expenditures in several countries in the region (5, 6). Cost control is an essential consideration in healthcare provision, and countries worldwide have applied various health economic principles, tools, and techniques to identify measures for health policy decision-making (7, 8).

Over the last 50 years, health economics research and its applications have gained much recognition worldwide (9). This recognition has begun to emerge in Africa, mainly due to concerns regarding healthcare expenditures, which have grown enormously across all public and private sectors (10–12). Previous empirical research in health economics in Africa has revealed the catastrophic impact of healthcare expenditure on households and how it has led to the underutilization of healthcare services (4, 12). The devastating effects of healthcare spending at the household level, coupled with burgeoning problems of shortages of essential medicine, poor quality of services, and the incessant migration of skilled primary healthcare workers to Western countries, have continued to render the supply side of African healthcare systems ineffective (11, 13). All these scenarios negate the main principle of Universal Health Coverage (UHC), which focuses on equitable access to and use of quality and effective health services without financial hardship (13, 14). Many reforms have been implemented, including the elimination of user fees, health insurance coverage, and results-based financing, to improve the health financing sector and help eradicate the challenges preventing full-scale UHC (15, 16). Despite these efforts, coverage of all forms of health insurance including community-based health insurance (CBHI) is limited with only approximately 2% of working adults in Africa being insured (17, 18). This situation poses a significant challenge and impedes access to quality healthcare for millions of individuals in the region. Health technology assessment (HTA), commonly used to scrutinize the adoption of new health technologies for clinical and cost-effectiveness in western countries has gradually found its way into African healthcare systems (19). This is a good development as it is now widely recognized as crucial to address the out-of-stock phenomenon that is one of the main features of public healthcare facilities in resource-limited settings (15, 19).

This Research Topic has attracted five significant contributions, four of which have been successfully published. These studies delve into crucial aspects of health economics in Africa, shedding light on important issues and providing valuable insights.

The first contribution is entitled “*The impact of community-based health insurance on household's welfare in Chilga District, Amhara Regional State, Ethiopia*”. This study examined the impact of government-sponsored Community-based health insurance (CBHI) on household welfare, focusing on its effectiveness in reducing catastrophic healthcare expenditures. The findings of the probit model revealed that Level of education, access to credit, living with a chronic disease, insurance premium, awareness of health insurance, distance to health services, and health service waiting times are significant determinants of enrollment in CBHI. Finally, the authors provided recommendations on how to improve service utilization, reduce per-capita healthcare expenditure, and increase per capita consumption. These recommendations includes expansion and accessibility of CBHI schemes at minimum premium, offering credit, strengthening education, establishing nearby health facilities, and efficient service provision (Asfaw et al.).

The second contribution is entitled “*Cost-effectiveness of community diabetes screening: application of Akaike information criterion in rural communities of Nigeria*”. The study examined the ability to combine biochemical and anthropometric parameters and orodental disease indicators (ODIs), precursors of periodontitis, to generate models for DM prediction using the Akaike information criterion (AIC), to ascertain the best model fit and to validate the health economics of diabetes screening in 433 subjects in Ndokwa communities of Nigeria. The results demonstrated that the cost of identifying < 2 new subjects with hyperglycemia in 1,000 people was ≥NGN 300,000 ($716). This cost of general screening for diabetes in rural communities may appear excessively high and challenging in terms of health economics. The analyses generated a total of 4,125 models. AIC modeling indicates that the FBG test is the best model (AIC = 4), and the least is a combination of random blood sugar + waist circumference + hip circumference (AIC ≈ 34). Models containing ODI parameters had AIC values >34, and consequently were deemed to be not recommendable. Overall, the study highlighted that ODIs have a low probability of predicting DM and thus need to be used with caution. Nonetheless, the authors concluded that adopting predictive models involving AIC is valuable in terms of cost-benefit and cost-effectiveness for healthcare consumers, favoring health economics (Anyasodor et al.).

The third contribution is entitled “*Assessing the potential of HTA to inform resource allocation decisions in low-income settings: the case of Malawi*”. This study investigated the need for wider use and the role of HTA in low-income countries, focusing on Malawi, a country with one of the lowest GDPs per capita. Financial resources available for health care are particularly scarce, amounting to only $39.5 per person annually. The authors developed a framework to classify the leading decisions on health technologies within health systems. The framework covers parameters such as identifying and prioritizing technologies for detailed assessment, deciding whether to adopt an intervention, assessing alternative investments for implementation and scale-up, and undertaking further research activities. This study was the first attempt to explore the feasibility of introducing HTA methods in highly resource-constrained health systems and how they could produce tangible results. The study acknowledged that difficulties such as the scarcity of resources, capacity, and data may impact the operationalization of HTA in resource-limited settings in general. However, the authors suggested that effective use of the Global Health Cost Effectiveness Registry, international collaboration, and capability building through the sharing of analytical capacity, resources, and expertise among LMICs would go a long way toward mitigating the resource limitations of HTA in these settings (Ramponi et al.).

The fourth contribution is “*Effectiveness and impact of community-based Health insurance on Health Service Utilization in Northwest Ethiopia: a quasi-experimental evaluation*”. The article evaluated the effectiveness of the CBHI program toward health services utilization and its impact in northwest Ethiopia using the effectiveness and impact dimensions of the Organization for Economic Cooperation and Development framework. The evaluation found 1.3 visits per capita per year of health service utilization among CBHI enrollees, an increase of 6.9 percentage points (ATT = 0.069; 95% CI: 0.034, 0.114). However, despite the improvement in utilization, the authors documented the existence of three challenges: (i) shortage of human resources, (ii) out-of-stock drugs and medical supplies, and (iii) long waiting times for services and reimbursement claims. They concluded that these challenges limit the program's success in achieving the projected health service utilization threshold and fall short of the WHO recommendation (Fetene et al.).

## Concluding remarks

All of the studies included in this Research Topic have, in one way or another, provided insights into the adoption and core challenges of healthcare financing, access to healthcare, and the evolving adoption of health technology assessment, primarily in Africa. The research summarized in this editorial reveals that significant progress has been made in the adoption of health economics tools, their sub-disciplines, and their implications for the UHC research agenda in some African countries. African research on health economics, particularly CBHI, is enormous and growing. The findings of these studies indicate that CBHI holds the promise of unrestricted access to and utilization of healthcare and preventive and curative care services across the board. Indeed, the results suggest that the availability of health insurance coverage could serve as a safety net and provide protection against catastrophic healthcare costs for households. Another important feature of CBHI as reported in one of the studies is the suggestion that the government (Ministry of Health) and concerned bodies (such as NGOs) should expand the coverage and accessibility of CBHI schemes, create awareness about CBHI in society, and subsidize the premium costs for people with low incomes. Both of these observations have policy consequences and should be replicated in other settings. Cost-effectiveness analysis within the framework of Health Technology Assessment (HTA) is critical. It could serve as a good policy tool and help guide the policymakers on interventions that need focusing to achieve optimal health benefits for the general population. This is particularly important in resource-limited settings where budget allocations for healthcare and the provision of healthcare are scarce and financial sustainability and future-proofing of life-saving interventions are needed. The study on HTA in Malawi is novel and shows how it could be applied in the entire African setting. Despite its novelty, the study stressed the importance of international collaboration and the use of a cost-effectiveness registry to ensure the robustness of the analysis for policy purposes. In summary, the findings of the studies mentioned in this editorial highlight the evolution, importance, and relative contributions of health economics to healthcare utilization, financing, and health policy in Africa. All these empirical contributions, taken together, have promising implications for future research in health economics in Africa. Thus, the sharing of ideas between researchers in different sub-disciplines of health economics in Africa and international collaborations with their counterparts worldwide would be of immense benefit in driving excellence in health economics research and policy in the region.
